# A Darker Shade of Love: Machiavellianism and Positive Assortative Mating Based on Romantic Ideals

**DOI:** 10.5964/ejop.v12i1.1007

**Published:** 2016-02-29

**Authors:** Tamás Ináncsi, András Láng, Tamás Bereczkei

**Affiliations:** a Department of General and Evolutionary Psychology, University of Pécs, Pécs, Hungary; b Department of Personality, Development and Clinical Psychology, University of Pécs, Pécs, Hungary; Aalborg University, Aalborg, Denmark

**Keywords:** Machiavellianism, romantic ideals, partner preferences, positive assortative mating, Ideal Standards Model, Big Five model of personality

## Abstract

Machiavellianism is a personality trait that is characterized by manipulative and exploitative attitude toward others, lack of empathy, and a cynical view of human nature. In itself or as part of the Dark Triad it has been the target of several studies investigating romantic relations. Nevertheless, the relationship between Machiavellianism and romantic ideals has not been revealed yet. An undergraduate sample of 143 (92 females) with an average age of 19.83 years (SD = 1.51 years) filled out self-report measures of Machiavellianism (Mach-IV Scale) and romantic ideals (Ideal Standards Scale and NEO-FFI-IDEAL). According to our results, Machiavellianism correlated negatively with the importance of partner’s warmth-trustworthiness, extraversion, openness, agreeableness, and with the importance of intimacy and loyalty in their ideal relationships. Machiavellianism correlated positively with the ideal partner’s possession over status and resources. Explorative factor analysis revealed three components of ideal partner’s characteristics. Machiavellianism loaded significantly on two out of three components. Results are discussed with regard to Ideal Standards Model and the Big Five model of personality.

## Machiavellian Personality and Close Relationship

Personality is a stable and permanent disposition accompanied by an individual pattern of interactions with the environment (Carver & Scheier, 2008). The literature has often discussed Machiavellianism as a personality trait or a complex of personality dimensions (Rauthmann & Will, 2011). In their pioneering work, Christie and Geis (1970) described Machiavellians as manipulative and cynical people who follow utilitarian morals. In Bakan’s bipolar model of social orientation, Machiavellians are characterized by agency and progress rather than connectedness to others and the community (Bakan, 1966). In Wiggins’ interpersonal circumplex that includes the two principal dimensions of love and dominance, Machiavellianism falls into the quadrant including traits such as callous - cold-hearted, self-seeking and dominant (Gurtman, 1992; Jones & Paulhus, 2011; Wiggins, 1985). Regarding the 16 bipolar trait dimensions proposed by Cattell, Eber, and Tatsuoka (1970), Machiavellianism may be delineated by a combination of attributes such as dominant, self-seeking - utilitarian, secretive, suspicious, norm-avoiding, retiring, thinking in concrete and pragmatic terms as well as emotionally unstable and anxious in the context of close relationships (Ináncsi, Láng, & Bereczkei, 2015).

Other personality psychologists have argued that the principal dimensions of personality are defined relatively accurately by the five personality traits known as the Big Five (McCrae & Costa, 1997). Previous research has revealed that Machiavellianism showed a negative relationship with agreeableness and conscientiousness (Austin, Farrelly, Black, & Moore, 2007; Jakobwitz & Egan, 2006; Lee & Ashton, 2005; Paulhus & Williams, 2002; Vernon, Villani, Vickers, & Harris, 2008) as well as with openness to new experience (Rauthmann, 2012) while it was positively related to neuroticism (Austin et al., 2007; Jakobwitz & Egan, 2006; Vernon et al., 2008). Research has found no significant relationship between extraversion and Machiavellianism. This is possibly because the dimension of extraversion is polarized along emotional stability. Extraversion accompanied by emotional instability is often conceptualized as striving for power and social dominance that are defining characteristics of Machiavellians. At the same time, extraversion associated with emotional stability – self-confidence (McHoskey, 2001), self-disclosure (Brown & Guy, 1983; Domelsmith & Dietch, 1978), the ability to experience pleasure (Egan, Chan, & Shorter, 2014) – is not characteristic of the Machiavellian personality.

Previous studies on Machiavellianism and close relationship focused on several aspects of interpersonal functioning. Christie and Geis (1970) in their pioneering work on Machiavellianism have already described the reserved and manipulative relational attitude of Machiavellian individuals. Since then Machiavellianism has been found to be connected to pragma love style (Jonason & Kavanagh, 2010), low levels of intimacy and commitment in the relationship (Ali & Chamorro-Premuzic, 2010), distrust towards the partner (Ináncsi et al., 2015), low emotional intelligence (Austin et al., 2007), low level of empathy (Andrew, Cooke, & Muncer, 2008), dismissive and fearful attachment styles (Gillath, Sesko, Shaver, & Chun, 2010; Ináncsi et al., 2015; Jonason, Lyons, & Bethell, 2014) destructive relational strategies (Pilch, 2012), short-term mate choice (Holtzman, 2013) and unrestricted sociosexuality (McHoskey, 2001). However, studies on the relationship between Machiavellianism and romantic ideals are still wanting.

### Assortative Mating Based on Romantic Ideals

According to the theory of assortative mating, people choose partners based on certain character traits (Thiessen & Gregg, 1980). Such choice is necessarily discriminative since one seeks for a partner who best meets one’s goals, needs, demands and expectations. Romantic ideals are organized around the person’s goals, and the preferred and expected character traits gain meaning and significance through the underlying goals. One’s partner ideal may be regarded either as one’s current concerns (Klinger, 1977), or as one’s personal projects (Little, 1983, 1989) since romantic ideals are personal conceptual-imaginative constructs (reference values, goal concepts) that determine precisely the optimal or desirable characteristics of a partner or relationship. Field theory proposes that one’s ideal partner and relationship are currently brought into the focus of one’s mind (Lewin, 1939).

Ideals are most similar to subjective values, attitudes or preferences (Simpson, Fletcher, & Campbell, 2001) and they determine expectations towards the self, the partner and the relationship (Campbell, Simpson, Kashy, & Fletcher, 2001; Fletcher, Simpson, Thomas, & Giles, 1999; Simpson et al., 2001). Previous research has revealed that one’s self-perception is related to one’s conception of the ideal partner (Murray, Holmes, & Griffin, 1996). For example, if one rates oneself high on a certain attribute, then one will also hold higher expectations towards one’s partner on the same dimension (Campbell et al., 2001; Fletcher, Simpson, & Thomas, 2000). Figueredo et al. (2006) revealed moderately positive correlations between the self and the ideal partner. They found that individuals seek for a mate being similar to them to a certain extent while they also seek for a partner who is somewhat more conscientious, more extraverted, more agreeable and emotionally less unstable than they themselves are.

Romantic ideals formulate specific expectations and they serve as a reference frame, a set of anchor points or standards (Fletcher et al., 1999). Ideals influence major relationship-related decisions and govern assortative mating through the appraisal of the partner and the relationship. During assortative mating, individuals assess each other by means of perceptual, emotional and cognitive mechanisms and continuously make comparisons between the partner’s perceived traits and the ideal image stored in their memory (Overall, Fletcher, & Simpson, 2006). Appraisal is based on three specific ideal dimensions: „(1) the prospective partners’ capacity for intimacy and commitment, (2) their attractiveness and general health, and (3) their social status and resources“ (Simpson, Fletcher, & Campbell, 2001, p. 91).

The process of ideal formation may be focused, on the one hand, on internal factors, that is, on directly not observable intra-individual traits and qualities (e.g. how much the partner is warm-hearted, commited and trustworthy). On the other hand, they may be focused on external factors which, being extra-individual, are more objective and more observable (e.g. status, material assets, physical attractiveness). Partner preferences vary according to the type of relationship. Internal attributes have much more importance in the development and stability of a long-term relationship while external characteristics gain more significance in short-term relationships (Campbell et al., 2001; Fletcher et al., 1999; Fletcher, Tither, O’Loughlin, Friesen, & Overall, 2004).

Mate choice as a psychological event potentially involves several dilemmas and contradictions due to the uncertainty of the subjective estimations of the partner’s warm-heartedness and reliability. Since partners are not able to directly read one another’s minds, internal qualities can only be inferred from external signals. Consequently, situations of decision-making in mate choice are poorly structured, characterized by “bounded rationality” (Simon, 1972, 1982). People cannot predict how much their chosen partners will be reliable or cooperating, how healthy they are and what amount of resources they are willing to invest in parenting offsprings. They do not know all important details of the partner, therefore in most cases they can only rely on intuition, presumptions, anticipation, ambiguous cues and seeming qualities (Goffman, 1959). Moreover, this uncertainty and unpredictability further increases during mate choice when partners attempt to manipulate and deceive one another by intentionally communicating false signals and information, as is often the case. Previous research has demonstrated that the Mach scale negatively correlates with the faith put in humanity and with the belief that most people are trustworthy and unselfish (Christie & Geis, 1970). This result also points out that Machiavellians perceive an external locus of control regarding internal factors. In cases of Machiavellians such a high level of uncertainty and distrust felt towards the partner and the relationship may result in negative expectations on the dimensions of the warm-heartedness - trustworthiness - intimacy romantic ideals (Ináncsi et al., 2015).

### Machiavellianism and Positive vs. Negative Assortative Mating

People develop idiosyncratic conceptions of the character traits their ideal partner should have in a close relationship. Depending on their preferences, they judge certain character traits as more important than others. In selecting their romantic partners, humans – irrespective of their culture – prefer warm, trustworthy and loyal partners (Buss, 1989). Fletcher et al. (1999, 2000) found in their studies that trust was consistently rated highest while Cottrell et al. (2007) have demonstrated that people regard trustworthiness as the primary value. Likewise, the most popular attributes according to subjects’ ratings of Anderson´s 555 adjectives derived from dictionary entries are “sincere, honest, understanding, loyal, true and trustworthy” while on the negative pole of the scale are “malevolent, unreliable, deceitful and lying” (Anderson, 1968). Considering the Big Five traits of personality, people regard such partners as ideal who are agreeable, extraverted, conscientious and emotionally stable (i.e. less neurotic) (Figueredo et al., 2006; Zentner, 2005).

There are at least two main paradigms of partner selection and both can be evidenced with previous research to be relevant to the partner selection of Machiavellians. On the one hand, similarity in level of affiliation, intimacy needs and personality traits has particular importance in ideal standards and mate choice. Previous studies provided countless evidence supporting the similarity hypothesis (Berscheid & Reis, 1998; Buss, 1985, 1999; Kandel, 1978; Markey & Markey, 2007; Wetzel & Insko, 1982). In this sense, people would maintain an ideal of their romantic partner, whose personality is similar to their own. This means that people choose mates in a positive assortative manner regarding personality traits. Evidently, this is because they hold that the similarity between themselves and their ideal partner is crucial for the maintenance and development of a close relationship. Several authors emphasize the complex relationship between self-perception and models of the ideal partner (Fletcher et al., 1999; Simpson et al., 2001). Meyer and Pepper (1977) pointed out in particular that partners’ balanced level of affiliation was an essential condition of happiness and satisfaction in a relationship. Accordingly, a Machiavellian individual with low affiliative abilities potentially should have the most harmonious relationship with another, emotionally reserved and distant high Mach person. Novgorodoff (1974) confirmed such preference of high-Mach women in a laboratory study. These high-Mach women chose men who scored even higher on a measure of Machiavellianism than themselves.

On the other hand, several studies confirmed negative assortative mate choice, i.e., choosing romantic partners based on the complementarity of traits and needs (Wiggins, 1979; Winch, 1958). Accordingly, it is also possible that a dominant high-Mach who demands attention can form the most stable – though dysfunctional and asymmetric – relationship with a submissive and permissive low-Mach partner who he or she can manipulate and exploit (Buss, 1984; Carson, 1969). Touhey (1977) found evidence for this negative assortative choice in his study on attitude similarity. He found that high-Mach individuals were more attracted to those who were the least similar to them.

### Machiavellianism and Personal Goals

People regard such romantic partners as ideal with whom they can realize their relationship goals (Fletcher et al., 1999). Since life is a course of realizing long-term and short-term interests and goals, relationship goals take the form of long-term and short-term strategies accordingly. Regan and Joshi (2003) also use the distinction between a “long-term romantic partner” and a “short-term sexual partner”. Borrowing the notion proposed by Dawkins (1976), people may follow the “strategy of family happiness” in so far as they take care of their offsprings or, alternatively, they can follow the feminine woman - masculine man strategy if they strive to increase the number of sexual contacts with the opposite sex. The literature reflects that Machiavellians prefer the latter: they follow a short-term mating strategy and they are inclined to promiscuity (Holtzman, 2013; McHoskey, 2001).

In investigating personal goal orientations, McHoskey (1999) found that Machiavellians were more willing to pursue extrinsic, material goals (e.g., money, fame) than intrinsic, relational goals such as spending time in community or with family. Accordingly, Jonason and Kavanagh (2010) found in their study that a more pronounced Machiavellian attitude showed the highest correlation to pragma love style. Lovers of this style are rational and realistic rather than romantic (Lee, 1973, 1977). Durkin´s (Christie & Geis, 1970) theory of encounter versus cognitive exchange of views also support that Machiavellian individuals are likely to ‘encounter blind’. It means that in interactions with others, high-Mach persons are unable to spontaneously disclose themselves, to be emotionally attuned to others and to become identic with common goals of the group or dyad. They rather tend to interact in a planned way that enables them to use interaction partners as a means to reaching their goals. Accordingly high Machs focus on goals governed by self-interest than form emotionally significant relationships with others.

## Aims of Our Study, Hypotheses

Empirical research may only provide information on assortative mating by studying and measuring accurately definable dimensions such as preferred attributes and personality traits, partner preferences and romantic ideals. We also chose this approach in the present study. The aim of our study was to investigate potential relationship between Machiavellianism and characteristics of their romantic partners using the Ideal Standard Model (Fletcher et al., 1999, 2000) and a five-factor theory of personality (McCrea & Costa, 1990).

We tested the following hypotheses:

1. Based on Machiavellians’ high level of uncertainty and distrust felt towards the partner and the relationship, high-Mach individuals were expected to conceive of an ideal partner as being less demanding, less emotional and less committed to the relationship. This would be reflected in such personality characteristics of the ideal partner as relatively low warmth, low agreeableness, and low conscientiousness, and in the ideal of a less intimate and loyal romantic relationship.
2. Given that Machiavellian individuals prefer short-term romantic relationships (Holtzman, 2013; McHoskey, 2001), we expected them to choose an ideal partner with traits that are important in short-term relationships. So, we expected high-Mach individuals – relative to their low-Mach peers – to prefer more attractive romantic partners with higher status.

Without formulating any particular hypothesis, we also wanted to reveal, if there were any latent dimensions (‘personality types’) underlying romantic partner personality traits and self-reported Machiavellianism.

## Method

### Sample and Procedure

Our sample consisted of 143 university students (92 females) studying law. Average age of the participants was 19.83 years of age (*SD* = 1.51; Min = 18; Max = 25). After giving their informed consent, participants filled out a booklet in groups of 30 to 40 depending on the size of the class. The booklet consisted of questions regarding basic demographical data and the measures presented below.

### Measures

#### Mach-IV Scale (Christie & Geis, 1970)

This 20-item instrument measures the agreement with Machiavellian attitudes. Participants had to indicate their level of agreement with statements (e.g., ‘The best way to handle people is to tell them what they want to hear’) on a 7-point Likert-scale. Although in several studies three or four subscales are used, we used the Mach-IV Scale as a unidimensional measure in this study. Internal reliability for this scale in our study proved to be excellent (Cronbach α = .81).

#### Ideal Standards Scales (Fletcher et al., 1999)

This measure consists of three parts including 46 adjectives that describe the ideal romantic partner, the ideal romantic relationship, and self as romantic partner. In the first and second parts, participants had to rate the importance of characteristics referring to the ideal romantic partner on three dimensions (warmth-trustworthiness, attractiveness-vitality, and possession over or potential to possess status-resources), and characteristics of the ideal romantic relationship on two dimensions (intimacy-loyalty, passion). In the third part, self-perception is evaluated on the same dimensions as the ideal romantic partner. All ratings were given on a 7-point Likert scale. Cronbach αs of the scales ranged between .72 and .91.

#### NEO-FFI-IDEAL (Figueredo, Sefcek, Asquez, et al., 2005)

This scale measures traits of the ideal partner on the five principal factors of the personality (neuroticism, extraversion, openness to experience, agreeableness and conscientiousness) according to the Big Five theory (McCrea & Costa, 1990). Participants indicated the importance of personality characteristics of the ideal partner on a 5-point Likert scale. Internal reliability of the factors were sufficient (.71 < Cronbach αs < .89).

### Statistical Analyses

For statistical analysis, we used SPSS 17.0 for Windows software package. Besides descriptive statistics, we used Pearson’s correlation to investigate the relationship between Machiavellianism and romantic ideal variables. We also used principal component analysis (PCA) with Varimax rotation to reveal complex ‘personality types’ of ideal romantic partners that might be associated with Machiavellianism.

## Results

We used Pearson’s correlation to reveal relationship between Machiavellianism scores, Ideal Standards Scale scores, and NEO-FFI-IDEAL scores partner and relationship variables. Results and descriptives of the variables are presented in Table 1. Weak to medium strength significant negative correlations were found between Mach-IV scores and scores on warmth-trustworthiness of the ideal partner, intimacy-loyalty in the ideal relationship, agreeableness, extraversion, openness and of the ideal romantic partner. A weak but significant positive correlation was also found between Machiavellianism and ideal partner’s possession over status-resources.

**Table 1:** Results of Pearson’s Correlations Between Mach-IV Scores, Ideal Partner Scale Scores, and NEO-FFI-IDEAL Scores with Descriptives (M ± SD) in the Diagonal Cells.

		Ideal Partner Dimensions			Ideal Relationship Dimensions		Self Dimensions			NEO-FFI-IDEAL Dimensions				
Measure	1	2	3	4	5	6	7	8	9	N	E	O	A	C
1. MACH-IV	97.72±11.67													
2. Warmth-trustworthiness	-.241**	6.24±.78												
3. Attractiveness-vitality	.022	.131	5.45±.72											
4. Status-resources	.214*	.065	.322**	5.05±1.15										
5. Passion	.052	.223**	.489**	.263**	5.72±.80									
6. Intimacy-loyalty	-.269**	.786**	.118	.170*	.173*	6.23±.79								
7. Warmth-trustworthiness	-.143	.616**	.184*	.105	.290**	.524**	5.60±.86							
8. Attractiveness-vitality	.147	.058	.580**	.425**	.439**	.091	.253**	5.09±.96						
9. Status-resources	.151	.061	.388**	.531**	.328**	.041	.122	.566**	5.57±.95					
N	.133	-.055	-.173*	-.091	-.058	-.047	-.101	-.175*	-.207*	2.17±.49				
E	-.240**	.244**	.243**	.056	.232**	.193*	.243**	.139	.138	-.256**	3.69±.39			
O	-.189*	.131	.214*	-.174*	.069	.080	.115	.087	.029	-.221**	.255**	3.44±.55		
A	-.423**	.424**	.030	-.072	-.076	.445**	.278**	-.059	-.040	-.283**	.293**	.235**	3.65±.58	
C	-.125	.343**	.066	.212*	.071	.374**	.309**	.092	.158	-.359**	.222**	.172*	.263**	4.20±.45

*_Note._*_N = Neuroticism; E = Extraversion; O = Openness; A = Agreeableness; C = Conscientiousness._

_*__p__ < .05. **__p__ < .01._

To find out whether there are certain types of ideal partners that are associated with Machiavellianism, principal component analysis (with Varimax rotation) was run on Mach-IV scores and variables connected to the ideal partner (i.e., three ideal partner dimension scores from the Ideal Standards Scale and five ideal partner personality dimension scores from NEO-FFI-IDEAL). Results are presented in Table 2. Analysis resulted in three components with eigenvalues over 1. We interpreted component 1 with the highest loadings for warmth-trustworthiness and agreeableness as ‘Miss / Mr. Right’, the perfect choice for a committed, devoted, long-term relationship. Machiavellianism loaded negatively (-.53) on this component. Component 2 described an adventurous, attractive and socially charming ideal partner. This component was labeled ‘Playmate’. Machiavellianism loaded low (-.15) on this component. Last, possession over status and resources, attractiveness-vitality, and Machiavellianism loaded high on Component 3. This component was labeled ‘Top Dog’, because this ideal partner is powerful and dominant. Machiavellianism loaded positively (.50) on this component. It is important to note that attractiveness-vitality loaded heavily on both components ‘Playmate’ and ‘Top Dog’. Based on the context, attractiveness was interpreted as the halo characteristic of a socially well-liked interaction partner (e.g., Dion, Berscheid, & Walster, 1972) in the case of ‘Playmate’, whereas attractiveness was interpreted as a means of power and manipulation (e.g., Chaiken, 1979; Hawley, Johnson, Mize, & McNamara, 2007) in the case of ‘Top Dog’.

**Table 2:** Results of Principal Components Analysis (With Varimax Rotation) of Machiavellianism and Ideal Partner Variables.

	Component		
	1. Miss / Mr. Right	2. Playmate	3. Top Dog
Warmth-trustworthiness	.770	.024	.092
Agreeableness	.722	.222	-.250
Conscientiousness	.612	.222	.338
Machiavellianism (self)	-.533	-.154	.500
Openness to experience	.042	.713	-.352
Attractiveness-vitality	-.082	.650	.413
Neuroticism	-.224	-.591	-.108
Extraversion	.328	.574	-.024
Status-resources	.100	.067	.864
Eigenvalue	2.56	1.53	1.09
Explained Variance (%)	21.67	19.32	16.55

## Discussion

This study was aimed at exploring Machiavellian individuals’ romantic ideals, their expectations towards partners and relationships as defined by their ideal formation processes and the resulting relationship goals that they strive to realize. A further aim of the study was to establish whether Machiavellians, when choosing a mate assortatively based on personality, prefer an ideal partner similar to them or they prefer a partner who has complementary attributes. Finally, we aimed to reveal whether there are specific latent personality types preferred by Machiavellian individuals as romantic partners.

According to previous results, Machiavellians learn during childhood that the significant others are dismissive, unhelpful and unreliable (Jonason et al., 2014; Kraut & Lewis, 1975; Láng & Lénárd, 2015; Ojha, 2007; Touhey, 1973). Such early experience prevents them from developing elaborate knowledge structures and models of a relationship based on warmth, intimacy, mutual acceptance and trust. Since relationship patterns developed in childhood remain active in adulthood (Hazan & Shaver, 1987), Machiavellians as adults are less willing to hold such demands towards their partner due to their negative expectations. Their ideals of internal character traits remain under-represented: the lack of experience of warmth results in the lack of need for warmth. In this way, Machiavellians’ internal mental working models developed in early childhood have direct influence on their idealization processes as well.

The results of our study have partly confirmed our hypotheses and they are consistent with the results of the above presented studies. In harmony with the prediction of the first hypothesis, Machiavellians expected less warm-heartedness, trustworthiness, extraversion, openness and agreeableness from their ideal partner. This is possibly because they themselves lack such intellectual abilities (Wastell & Booth, 2003). High scores on Mach-IV also deemed the intimacy of their ideal relationship and the loyalty in this relationship less important than those who had low Mach-IV scores. These results indeed reflect that neither having a warm-hearted, loyal and trustworthy partner is Machiavellians’ primary relationship goal, nor are they interested in building long-term partner relationships characterized by intimacy and loyalty (Ali & Chamorro-Premuzic, 2010; Dussault, Hojjat, & Boone, 2013). This is not surprising given that Machiavellians are characterized by dismissive and fearful attachment styles (Gillath et al., 2010; Ináncsi et al., 2015; Jonason et al., 2014) as well as by a pragma love style (Jonason & Kavanagh, 2010; Lee, 1973, 1977) whose principal feature is the avoidance of emotions and intimacy. A possible consequence of this is that avoidance of intimacy becomes the focal theme for both Machiavellians and their ideal partners.

Results obtained for Hypothesis 2 provide the answer to the question what Machiavellians’ major relationship goals are. Results only partly confirmed this hypothesis. Machiavellianism correlated positively with the ideal partner’s status and resources while no significant correlation was obtained between Machiavellianism and the importance of the partner’s attractiveness and vitality. The results suggest that Machiavellians’ primary relationship goal is finding a partner who possesses a large amount of financial and other material resources and a prominent status in the social hierarchy. Due to their pragmatic attitude, they appraise their partners in term of constructs related to the partner’s utility for the Machiavellian’s individual progress. Our results are consistent with those obtained by McHoskey (1999) who found that Machiavellians hold extrinsic material goals (e.g. status and resources) much more important than intrinsic relationship goals (community, family, romantic love). The presumable cause of this exclusive focus on external relationship factors for Machiavellians might be due to their feelings of uncertainty and distrust towards others and relationships in general. They conceive of intimacy as dangerous involving such a high level of personal risk which exceeds their own capacity. The perceived external control results in their being afraid of engaging in sincere close relationships in which partners disclose their genuine private self. Therefore Machiavellian individuals are unable to participate in the relationship for the pleasure of communion led by intrinsic motivation (Durkin, 1970) and they do not gain rewarding experience related to internal factors. Thus, in part by the perceived external control and in part by expected external rewards, their behaviour becomes externally controlled and extrinsically motivated, and eventually the relationship is reduced to a mere means of achieving external goals (material assets, status and sexual satisfaction). These results are also consistent with Machiavellians’ short-term mating strategies (Holtzman, 2013; McHoskey, 2001; Simpson & Gangestad, 1991) as well as with their fatalistic perspective on the future (Birkás & Csathó, 2015).

With regard to personality traits, Machiavellians assign little importance to such universally highly valued traits as extraversion, openness and agreeableness (Figueredo et al., 2005). Although self-confidence, spontaneity, activity, the ability to experience pleasure, intelligence, creativity, imaginativeness, curiosity, assertiveness, sociability and positive emotional orientation are generally highly valued during mate choice, Machiavellians do not find them attractive when thinking of an ideal partner. There are at least two possible interpretations of these results. First, introverted, rigid, emotionally cold and egocentric partners provide the opportunity to establish a relationship which involves a low level of need for intimacy. Second, more submissive, irresolute, adaptable and dependent partners are more easily influenced and more predictable. Such partners offer Machiavellians a feeling of control over the relationship which they hold particularly important (Christie & Geis, 1970).

Our results suggest that Machiavellians choose partners who are mostly similar to them in personality traits, with especial regard to the central dimension of relationships, that is, warm-heartedness, trustworthiness and intimacy. This is in line with the position of Meyer and Pepper (1977) who propose that a balanced level of affiliation between the partners is a basic condition of happiness in the relationship. Heider’s cognitive balance theory also suggests that people prefer partners who have similar attitudes and thereby reinforce their worldviews (Heider, 1946, 1958). According to Willi’s (1984) concept of collusion attraction between future partners is based on a mutual alarming theme, shared by both partners in order to be mastered together. The plausibility of this explanation is further strengthened by the results of Krueger, Moffitt, Caspi, Bleske, and Silva (1998) who found evidence for positive assortative mating for antisocial behavior in 360 couples.

Our results are also compatible with the explanatory framework of positive assortative mating based on personality (Arrindell & Luteijn, 2000). Machiavellians are characterized in the literature by low agreeableness, low conscientiousness, low openness, high neuroticism (Jakobwitz & Egan, 2006; Lee & Ashton, 2005; Paulhus & Williams, 2002; Rauthmann, 2012; Vernon et al., 2008) and theoretically low extraversion. According to our results, these characteristics are substantially the same as characteristics preferred by Machiavellian individuals in their ideal romantic partners.

Although our results did not confirm the expected relationship between Machiavellianism and the external factors of attractiveness and vitality, a relevant study found that Machiavellianism is in a positive relationship with physically attractive self-presentation (Holtzman & Strube, 2012). Consequently, Machiavellians do not expect their ideal partners but do expect themselves to present attractive physical appearance thereby making a favourable impression.

In revealing potential types of ideal partners, we found that Machiavellian individuals disliked the ‘Miss / Mr. Right’ type, who would be a perfect choice for a long-term, emotionally intense, and committed romantic relationship. Machiavellians seemed to prefer ‘Top Dogs’, attractive and vital romantic partners who possess considerable status and resources. At first glance, this result contradicts our result considering Machiavellians’ lack of preference for vitality and attractiveness in ideal romantic partners. In our opinion, attractiveness and vitality is not a matter of aesthetics for ‘Top Dogs’, rather a means of getting on in our more and more narcissistic society (Verhulst, Lodge, & Lavine, 2010).

These results lead to the conclusion that Machiavellians’ choice of a mate is primarily mediated by sensory information instead of psychological dimensions and the major aspects of their preferences are of external-material nature. In their relationships, they presumably orient themselves towards gaining highly valued external resources and social positions rather than mutual self-disclosure.

### Limitations and Further Directions

Our study has its limitations of course. Since our results are based on self-report data considering romantic ideals we must stay reserved considering the ecological validity of our study. Previous research proved (e.g., Swami & Barrett, 2011) that participants tend to report attitudes different from their actual, observed behavior in the domain of mate choice. So, further research might use couples’ reports of personality and Machiavellianism, and test the relationship of these constructs with actual marital satisfaction, or forms of dyadic behavior.

## References

[r1] Ali F. Chamorro-Premuzic T. (2010). The dark side of love and life satisfaction: Association with intimate relationships, psychopathy and Machiavellianism. Personality and Individual Differences, 48, 228–233. 10.1016/j.paid.2009.10.016

[r2] Anderson N. H. (1968). Likableness ratings of 555 personality-trait words. Journal of Personality and Social Psychology, 9(3), 272–279. 10.1037/h0025907 5666976

[r3] Andrew J. Cooke M. Muncer S. J. (2008). The relationship between empathy and Machiavellianism: An alternative to empathizing – Systemizing theory. Personality and Individual Differences, 44, 1203–1211. 10.1016/j.paid.2007.11.014

[r4] Arrindell W. A. Luteijn F. (2000). Similarity between intimate partners for personality traits as related to individual levels of satisfaction with life. Personality and Individual Differences, 28(4), 629–637. 10.1016/S0191-8869(99)00125-7

[r5] Austin E. J. Farrelly D. Black C. Moore H. (2007). Emotional intelligence, Machiavellianism and emotional manipulation: Does EI have a dark side? Personality and Individual Differences, 43, 179–189. 10.1016/j.paid.2006.11.019

[r6] Bakan, D. (1966). *The duality of human existence: Isolation and communion in Western man.* Boston, MA, USA: Beacon Press.

[r7] Berscheid, E., & Reis, H. T. (1998). Attraction and close relationships. In D. T. Gilbert, S. T. Fiske, & G. Lindzey (Eds.), *The handbook of social psychology* (4th ed.). Oxford, United Kingdom: Oxford University Press.

[r8] Birkás B. Csathó Á. (2015). Size the day: The time perspectives of the Dark Triad. Personality and Individual Differences, 86, 318–320. 10.1016/j.paid.2015.06.035

[r9] Brown E. C. Guy R. F. (1983). The effects of sex and Machiavellianism on self-disclosure patterns. Social Behavior and Personality, 11(1), 93–96. 10.2224/sbp.1983.11.1.93

[r10] Buss D. M. (1984). Marital assortment for personality dispositions: Assessment with three different data sources. Behavior Genetics, 14, 111–123. 10.1007/BF01076408 6721817

[r11] Buss D. M. (1985). Human mate selection. American Scientist, 73, 47–51.

[r12] Buss D. M. (1989). Sex differences in human mate preferences: Evolutionary hypotheses tested in 37 cultures. Behavioral and Brain Sciences, 12, 1–14. 10.1017/S0140525X00023992

[r13] Buss, D. M. (1999). *Evolutionary psychology: The new science of the mind.* Boston, MA, USA: Ally and Bacon.

[r14] Campbell L. Simpson J. A. Kashy D. A. Fletcher G. J. O. (2001). Ideal standards, the self, and flexibility of ideals in close relationships. Personality and Social Psychology Bulletin, 27, 447–462. 10.1177/0146167201274006

[r15] Carson, R. (1969). *Interaction concepts of personality.* Chicago, IL, USA: Aldine.

[r16] Carver, C. S., & Scheier, M. F. (2008). *Perspectives on personality* (7th ed.). Boston, MA, USA: Pearson / Allyn and Bacon.

[r17] Cattell, R. B., Eber, H. W., & Tatsuoka, M. M. (1970). *Handbook for the Sixteen Personality Factor Questionnaire.* Champaign, IL, USA: Institute for Personality and Ability Testing.

[r18] Chaiken S. (1979). Communicator physical attractiveness and persuasion. Journal of Personality and Social Psychology, 37, 1387–1397. 10.1037/0022-3514.37.8.1387

[r19] Christie, R., & Geis, F. L. (1970). *Studies in Machiavellianism.* New York, NY, USA: Academic Press.

[r20] Cottrell C. A. Neuberg S. L. Li N. P. (2007). What do people desire in others? A sociofunctional perspective on the importance of different valued characteristics. Journal of Personality and Social Psychology, 92, 208–231. 10.1037/0022-3514.92.2.208 17279846

[r21] Dawkins, R. (1976). *The selfish gene.* Oxford, United Kingdom: Oxford University Press.

[r22] Dion K. Berscheid E. Walster E. (1972). What is beautiful is good. Journal of Personality and Social Psychology, 24, 285–290. 10.1037/h0033731 4655540

[r23] Domelsmith D. E. Dietch J. T. (1978). Sex differences in the relationship between Machiavellianism and self-disclosure. Psychological Reports, 42, 715–721. 10.2466/pr0.1978.42.3.715

[r24] Durkin, J. E. (1970). Encountering: What low Machs do. In R. Christie & F. L. Geis (Eds.), *Studies in Machiavellianism.* New York, NY, USA: Academic Press.

[r25] Dussault M. Hojjat M. Boone R. T. (2013). Machiavellianism and dating: Deception and intimacy. Social Behavior and Personality, 41(2), 283–294. 10.2224/sbp.2013.41.2.283

[r26] Egan V. Chan S. Shorter G. W. (2014). The dark triad, happiness and subjective well-being. Personality and Individual Differences, 67, 17–22. 10.1016/j.paid.2014.01.004

[r27] Figueredo, A. J., Sefcek, J. A., Asquez, G., Brumbach, B. H., King, J. E., & Jacobs, W. J. (2005). Evolutionary personality psychology. In D. M. Buss (Ed.), *Handbook of evolutionary psychology.* Hoboken, NJ, USA: Wiley.

[r28] Figueredo A. J. Sefcek J. A. Jones D. N. (2006). The ideal romantic partner personality. Personality and Individual Differences, 41, 431–441. 10.1016/j.paid.2006.02.004

[r29] Fletcher G. J. O. Simpson J. A. Thomas G. (2000). Ideals, perceptions, and evaluations in early relationship development. Journal of Personality and Social Psychology, 79, 933–940. 10.1037/0022-3514.79.6.933 11138762

[r30] Fletcher G. J. O. Simpson J. A. Thomas G. Giles L. (1999). Ideals in intimate relationships. Journal of Personality and Social Psychology, 76, 72–89. 10.1037/0022-3514.76.1.72 9972554

[r31] Fletcher G. J. O. Tither J. M. O’Loughlin C. Friesen M. Overall N. (2004). Warm and homely or cold and beautiful? Sex differences in trading off traits in mate selection. Personality and Social Psychology Bulletin, 30, 659–672. 10.1177/0146167203262847 15155031

[r32] Gillath O. Sesko A. K. Shaver P. R. Chun D. S. (2010). Attachment, authenticity, and honesty: Dispositional and experimentally induced security can reduce self- and other-deception. Journal of Personality and Social Psychology, 98, 841–855. 10.1037/a0019206 20438228

[r33] Goffman, E. (1959). *The presentation of self in everyday life.* New York, NY, USA: Anchor Books.

[r34] Gurtman M. B. (1992). Trust, distrust, and interpersonal problems: A circumplex analysis. Journal of Personality and Social Psychology, 62, 989–1002. 10.1037/0022-3514.62.6.989 1619552

[r35] Hawley P. H. Johnson S. E. Mize J. A. McNamara K. A. (2007). Physical attractiveness in preschoolers: Relationships with power, status, aggression and social skills. Journal of School Psychology, 45(5), 499–521. 10.1016/j.jsp.2007.04.001

[r36] Hazan C. Shaver P. (1987). Romantic love conceptualized as an attachment process. Journal of Personality and Social Psychology, 52(3), 511–524. 10.1037/0022-3514.52.3.511 3572722

[r37] Heider F. (1946). Attitudes and cognitive organization. The Journal of Psychology, 21, 107–112. 10.1080/00223980.1946.9917275 21010780

[r38] Heider, F. (1958). *The psychology of interpersonal relations.* New York, NY, USA: John Wiley & Sons.

[r39] Holtzman N. S. (2013). Above and beyond short-term mating, long-term mating is uniquely tied to human personality. Evolutionary Psychology, 11(5), 1101–1129. doi:. 10.1177/147470491301100514 24342881 PMC10430001

[r40] Holtzman N. S. Strube M. J. (2012). People with dark personalities tend to create a physically attractive veneer. Social Psychological and Personality Science, 4, 461–467. 10.1177/1948550612461284

[r41] Ináncsi T. Láng A. Bereczkei T. (2015). Machiavellianism and adult attachment in general interpersonal relationships and close relationships. Europe's Journal of Psychology, 11(1), 139–154. 10.5964/ejop.v11i1.801 PMC4873099 27247647

[r42] Jakobwitz S. Egan V. (2006). The dark triad and normal personality traits. Personality and Individual Differences, 40, 331–339. 10.1016/j.paid.2005.07.006

[r43] Jonason P. K. Kavanagh P. (2010). The dark side of love: Love styles and the Dark Triad. Personality and Individual Differences, 49, 606–610. 10.1016/j.paid.2010.05.030

[r44] Jonason P. K. Lyons M. Bethell E. (2014). The making of Darth Vader: Parent-child care and the Dark Triad. Personality and Individual Differences, 67, 30–34. 10.1016/j.paid.2013.10.006

[r45] Jones, D. N., & Paulhus, D. L. (2011). Differentiating the Dark Triad within the interpersonal circumplex. In L. M. Horowitz & S. Strack (Eds.), *Handbook of interpersonal psychology: Theory, research, assessment, and therapeutic interventions* (pp. 249-269). New York, NY, USA: John Wiley & Sons.

[r46] Kandel D. B. (1978). Homophily, selection, and socialization in adolescent friendships. American Journal of Sociology, 84(2), 427–436. 10.1086/226792

[r47] Klinger, E. (1977). *Meaning and void: Inner experience and the incentives in people’s lives.* Minneapolis, Minnesota, MN, USA: University of Minnesota Press.

[r48] Kraut R. E. Lewis S. H. (1975). Alternate models of family influence on student political ideology. Journal of Personality and Social Psychology, 31, 791–800. 10.1037/h0076688

[r49] Krueger R. F. Moffitt T. E. Caspi A. Bleske A. Silva P. A. (1998). Assortative mating for antisocial behavior: Developmental and methodological implications. Behavior Genetics, 28(3), 173–186. 10.1023/A:1021419013124 9670593

[r50] Láng A. Lénárd K. (2015). The relation between memories of childhood psychological maltreatment and Machiavellianism. Personality and Individual Differences, 77, 81–85. 10.1016/j.paid.2014.12.054

[r51] Lee, J. A. (1973). *The colours of love: An exploration of the ways of loving.* Don Mills, Ontario, Canada: New Press.

[r52] Lee J. A. (1977). A typology of styles of loving. Personality and Social Psychology Bulletin, 3, 173–182. 10.1177/014616727700300204

[r53] Lee K. Ashton M. C. (2005). Psychopathy, Machiavellianism, and Narcissism in the Five-Factor Model and the HEXACO model of personality structure. Personality and Individual Differences, 38, 1571–1582. 10.1016/j.paid.2004.09.016

[r54] Lewin K. (1939). Field theory and experiment in social psychology: Concepts and methods. American Journal of Sociology, 44(6), 868–896. 10.1086/218177

[r55] Little B. R. (1983). Personal projects: A rationale and method for investigation. Environment and Behavior, 15, 273–309. 10.1177/0013916583153002

[r56] Little, B. R. (1989). Personal projects analysis: Trivial pursuits, magnificent obsessions, and the search for coherence. In D. Buss & N. Cantor (Eds.), *Personality psychology: Recent trends and emerging directions* (pp. 15-31). New York, NY, USA: Springer.

[r57] Markey P. M. Markey C. N. (2007). Romantic ideals, romantic obtainment, and relationship experiences: The complementarity of interpersonal traits among romantic partners. Journal of Social and Personal Relationships, 24, 517–533. 10.1177/0265407507079241

[r58] McCrea, R. R., & Costa, P. T., Jr. (1990). *Personality in adulthood.* New York, NY, USA: Guilford Press.

[r59] McCrae R. R. Costa P. T. Jr. (1997). Personality trait structure as a human universal. American Psychologist, 52, 509–516. 10.1037/0003-066X.52.5.509 9145021

[r60] McHoskey J. W. (1999). Machiavellianism, intrinsic versus extrinsic goals, and social interest: A self-determination theory analysis. Motivation and Emotion, 23, 267–283. 10.1023/A:1021338809469

[r61] McHoskey J. W. (2001). Machiavellianism and sexuality: On the moderating role of biological sex. Personality and Individual Differences, 31, 779–789. 10.1016/S0191-8869(00)00180-X

[r62] Meyer J. P. Pepper S. (1977). Need compatibility and marital adjustment in young married couples. Journal of Personality and Social Psychology, 35(5), 331–342. 10.1037/0022-3514.35.5.331 874737

[r63] Murray S. L. Holmes J. G. Griffin D. W. (1996). The benefits of positive illusions: Idealization and the construction of satisfaction in close relationships. Journal of Personality and Social Psychology, 70, 79–98. 10.1037/0022-3514.70.1.79

[r64] Novgorodoff B. D. (1974). Boy meets girl: Machiavellianism and romantic attraction. Personality and Social Psychology Bulletin, 1, 307–309. 10.1177/0146167274001001104

[r65] Ojha H. (2007). Parent-child interaction and Machiavellian orientation. Journal of the Indian Academy of Applied Psychology, 33(2), 285–289.

[r66] Overall N. C. Fletcher G. J. O. Simpson J. A. (2006). Regulation processes in intimate relationships: The role of ideal standards. Journal of Personality and Social Psychology, 91(4), 662–685. 10.1037/0022-3514.91.4.662 17014292

[r67] Paulhus D. L. Williams K. M. (2002). The dark triad of personality: Narcissism, Machiavellianism, and Psychopathy. Journal of Research in Personality, 36, 556–563. 10.1016/S0092-6566(02)00505-6

[r68] Pilch I. (2012). Machiavellianism and problem-solving strategies in a marriage relationship. The New Educational Review, 27(1), 324–336.

[r69] Rauthmann J. F. (2012). Towards multifaceted Machiavellianism: Content, factorial, and construct validity of a German Machiavellianism Scale. Personality and Individual Differences, 52, 345–351. 10.1016/j.paid.2011.10.038

[r70] Rauthmann J. F. Will T. (2011). Proposing a multidimensional Machiavellianism conceptualization. Social Behavior and Personality, 39(3), 391–403. 10.2224/sbp.2011.39.3.391

[r71] Regan P. C. Joshi A. (2003). Ideal partner preferences among adolescents. Social Behavior and Personality, 31(1), 13–20. 10.2224/sbp.2003.31.1.13

[r72] Simon, H. A. (1972). Theories of bounded rationality. In C. B. McGuire & R. Radner (Eds.), *Decision and organization* (pp. 161-176). Amsterdam, The Netherlands: North-Holland Publishing Company.

[r73] Simon, H. A. (1982). *Models of bounded rationality: Economic analysis and public policy* (Vol. 1). Cambridge, MA, USA: MIT Press.

[r74] Simpson, J. A., Fletcher, G. J. O., & Campbell, L. (2001). The structure and function of ideal standards in close relationships. In G. J. O. Fletcher & M. S. Clark (Eds.), *Blackwell handbook of social psychology: Interpersonal processes* (pp. 86-106). Malden, MA, USA: Blackwell.

[r75] Simpson J. A. Gangestad S. W. (1991). Individual differences in sociosexuality: Evidence for convergent and discriminant validity. Journal of Personality and Social Psychology, 60(6), 870–883. 10.1037/0022-3514.60.6.870 1865325

[r76] Swami V. Barrett S. (2011). British men’s hair color preferences: An assessment of courtship solicitation and stimulus ratings. Scandinavian Journal of Psychology, 52(6), 595–600. 10.1111/j.1467-9450.2011.00911.x 21883260

[r77] Thiessen D. D. Gregg B. (1980). Human assortative mating and genetic equilibrium: An evolutionary perspective. Ethology and Sociobiology, 1, 111–140. 10.1016/0162-3095(80)90003-5

[r78] Touhey J. C. (1973). Child-rearing antecedents and the emergence of Machiavellianism. Sociometry, 36(2), 194–206. 10.2307/2786566 4710175

[r79] Touhey J. C. (1977). Personality correlates of attraction in response to attitude similarity. European Journal of Social Psychology, 7(1), 117–119. 10.1002/ejsp.2420070110

[r80] Verhulst B. Lodge M. Lavine H. (2010). The attractiveness halo: Why some candidates are perceived more favorably than others. Journal of Nonverbal Behavior, 34(2), 111–117. 10.1007/s10919-009-0084-z

[r81] Vernon P. A. Villani V. C. Vickers L. C. Harris J. A. (2008). A behavioral genetic investigation of the Dark Triad and the Big 5. Personality and Individual Differences, 44, 445–452. 10.1016/j.paid.2007.09.007

[r82] Wastell C. Booth A. (2003). Machiavellianism: An Alexithymic perspective. Journal of Social and Clinical Psychology, 22, 730–744. 10.1521/jscp.22.6.730.22931

[r83] Wetzel C. G. Insko C. A. (1982). The similarity-attraction relationship: Is there an ideal one? Journal of Experimental Social Psychology, 18, 253–276. 10.1016/0022-1031(82)90053-1

[r84] Wiggins J. S. (1979). A psychological taxonomy of trait-descriptive terms: The interpersonal domain. Journal of Personality and Social Psychology, 37(3), 395–412. 10.1037/0022-3514.37.3.395

[r85] Wiggins, J. S. (1985). The interpersonal circle: A structural model for the integration of personality research. In R. Hogan & W. H. Jones (Eds.), *Perspectives in personality* (Vol. 1, pp. 1-47). Greenwich, CT, USA: JAI Press.

[r86] Willi J. (1984). The concept of collusion: A combined systemic-psychodynamic approach to marital therapy. Family Process, 23(2), 177–185. 10.1111/j.1545-5300.1984.00177.x 6734790

[r87] Winch, R. F. (1958). *Mate-selection: A study of complementary needs.* New York, NY, USA: Harper.

[r88] Zentner M. R. (2005). Ideal mate personality concepts and compatibility in close relationshps: A longitudinal analysis. Journal of Personality and Social Psychology, 89(2), 242–256. 10.1037/0022-3514.89.2.242 16162056

